# Impact of COVID-19 on Stroke Admissions and the Medical Care System in the Tokyo Metropolitan Area

**DOI:** 10.3389/fneur.2020.601652

**Published:** 2020-11-30

**Authors:** Takahiro Ota, Yoshiaki Shiokawa, Teruyuki Hirano

**Affiliations:** ^1^Department of Neurosurgery, Tokyo Metropolitan Tama Medical Center, Fuchu, Japan; ^2^Department of Neurosurgery, Kyorin University, Mitaka, Japan; ^3^Department of Stroke and Cerebrovascular Medicine, Kyorin University, Mitaka, Japan

**Keywords:** care system, COVID-19, Tokyo, stroke, thrombectomy

## Abstract

**Aims:** This study aimed to assess the number of patients with acute stroke seeking medical emergency care since the declaration of the state of emergency in the COVID-19 pandemic in the Tokyo metropolitan area of Japan.

**Methods:** In this combined retrospective and prospective multicenter survey, data on the numbers of hospital admissions due to acute ischemic stroke, of large vessel occlusion (LVO) cases, and of reperfusion therapies performed from February to July 2020, restrictions of the medical care system, and comprehensive stroke center (CSC) scale scores were collected in 19 stroke centers in Tokyo.

**Results:** In the survey period, 3,456 patients were admitted with acute stroke. There was a decrease in the number of admissions (−22%), LVO (−22%), thrombolysis (−6%), and thrombectomy (−23%) during the state of emergency, but the ratio of thrombectomy to LVO cases was not different. The acceptance of acute stroke cases by emergency transport and emergent operations in the central eastern area of Tokyo, was also significantly decreased to <50% and remains <60%. According to CSC scores, each hospital restricted their infrastructure or educational activities according to their medical resources. There was only one stroke case with COVID-19 (thrombectomy case) in all 3,456 patients in this study.

**Conclusion:** The COVID-19 pandemic had a major impact on stroke care in Tokyo, including stroke admissions and medical care systems, resulting in a significant reduction in thrombolysis and thrombectomy. The extent of the drop may be the result of the number of COVID-19 patients.

## Introduction

The COVID-19 pandemic declared by the World Health Organization (WHO) in March 2020 has challenged healthcare systems and societies worldwide. During the pandemic, there have been many reports that the number of stroke patients seen in the emergency department has dropped considerably, with a significant reduction in both thrombolysis and mechanical thrombectomy (MT) (1, 2).

On January 16, 2020, Japan reported its first case of COVID-19. A state of emergency was declared from April 7 to May 25 with a massive increase of patients. The declaration had a great impact on the stroke management system, especially on the emergent care of acute stroke. The largest number of patients with COVID-19 is found in the Tokyo metropolitan area, which is the capital of Japan. The Tokyo metropolitan area is highly populated and consists of 23 wards (the eastern half, with a population of about 8 million in an area of 619 km^2^), the Tama area (the western half, with a population of 4.3 million and an area of 1,160 km^2^), and the island regions. By July 30, the total number of COVID-19 cases was 10,408 (88.3%) in the 23 wards and 1,380 (11.7%) in the Tama area.

## Aims

The objective of this study was to assess and quantify the dynamics of admission of acute stroke cases and the number of reperfusion therapies performed during the COVID-19 pandemic and to estimate the real impact of the state of emergency declaration on the emergency stroke care system in the Tokyo metropolitan area in Japan.

## Methods

This was a multicenter, combined retrospective and prospective observational study involving a questionnaire survey. The questionnaire was sent, and data were collected every 2 weeks from all the hospitals of the Tokyo/tama-Registry of Acute endovascular Thrombectomy (TREAT) (3) between February 1 and July 31, 2020 (retrospectively to March 31 and prospectively from April). The participating facilities were 11 of 13 recanalization therapy-capable stroke centers in the Tama area and 8 of about 45 recanalization therapy-capable stroke centers in the 23 wards. The cases were grouped in three periods of 2 months each, according to the declaration of the state of emergency (from April to May) in Japan. All centers were asked to answer a short questionnaire about the following items: the number of acute stroke admissions, the number of patients with large vessel occlusion (LVO), the mean (2 weeks) number of MT and thrombolysis cases, the change in the comprehensive stroke center (CSC) score (4), and the quantitative restrictions of the medical care system (outpatient department, emergency visits, elective operations, emergent operations).

## Statistics

Descriptive statistics were used to compare the incidence of stroke admissions before and after the declaration of the state of emergency in Japan. Comparisons between groups were made using chi-squared tests for categorical variables, with *p*-values < 0.05 considered significant. Categorical data are expressed as the number of stroke admission, LVO cases, thrombolysis cases, and thrombectomy cases. All statistical analyses were performed using EZR (Saitama Medical Center, Jichi Medical University, Saitama, Japan), a graphical user interface for R (The R Foundation for Statistical Computing, Vienna, Austria) (5).

## Results

In total, 3,456 patients with acute stroke (2,354 in Tama and 1,102 in the 23 wards) presented to the participating hospitals (Table 1). Drops in the numbers of stroke cases, LVO, thrombolysis, and MT occurred during the state of emergency, but they did not occur homogeneously across the areas. The decreases in LVO and MT were greater in the 23 wards, but there were no significant differences in the decreases in the numbers of stroke admissions and LVO, thrombolysis, and MT cases compared to the pre-2 months before declaration of the state of emergency. One finding particularly worth mentioning is that there was only one stroke case (0.03%) with COVID-19 (an MT case) in all 3,456 patients in this study.

**Table 1:** Numbers of acute stroke admissions and large vessel occlusion, thrombolysis, and mechanical thrombectomy cases.

	**Pre (February, March)**	**Period of the state of emergency (April, May)**	**Post (June, July)**	***p***
**Acute stroke**				n.s.
Total	329	259 (78.7%)	276 (83.9%)	
Tama area	218	180 (82.6%)	191 (87.6%)	
23 wards	112	79 (70.5%)	86 (76.8%)	
**Large vessel occlusion**				n.s.
Total	45	35 (77.8%)	32 (71.1%)	
Tama area	28	24 (85.7%)	22 (78.6%)	
23 wards	18	11 (61.1%)	10 (55.6%)	
**Thrombolysis**				n.s.
Total	17	16 (94.1%)	16 (94.1%)	
Tama area	13	13 (100.0%)	11 (84.6%)	
23 wards	4	3 (75.0%)	5 (125%)	
**Thrombectomy**				n.s.
Total	26	20 (76.9%)	20 (76.9%)	
Tama area	14	13 (92.9%)	13 (92.9%)	
23 wards	13	7 (53.8%)	7 (53.8%)	
**Ratio of thrombectomy to total large vessel occlusion cases, %**				<0.05
Total	57.8	57.1	62.5	
Tama area	50	54.2	59.1	
23 wards	72.2	63.6	70	

*Values are mean numbers of admissions /2 weeks, n.s., not significant. The radio of period of the state of emergency and Post to Pre are shown (%)*.

## Quantitative Restrictions of the Medical Care System

Restrictions of the outpatient department, emergency visits, elective operations, and emergent operations are shown in Table 2.

**Table 2:** The mean number and percentage of hospitals with normal medical care systems.

	**Pre (February, March)**	**Period of the state of emergency (April, May)**	**Post (June, July)**	***p***
**Outpatient department**				not significant
Tama area	9.7 (88.6%)	3.0 (27.2%)	10.0 (91%)	
23 wards	7.2 (90.6%)	3.8 (46.9%)	7.0 (87.5%)	
**Elective operations**				*p* < 0.05
Tama area	9.5 (86.4%)	1.5 (13.6%)	8.0 (72.7%)	
23 wards	7.5 (93.8%)	2.0 (25%)	5.8 (71.9%)	
**Emergent operations**				*p* < 0.05
Tama area	11.0 (100%)	9.5 (86.4%)	10.3 (93.2%)	
23 wards	7.8 (96.9%)	4.5 (56.3%)	4.8 (59.4%)	
**Emergent transfer of acute stroke cases**				*p* < 0.05
Tama area	11.0 (100%)	9.0 (81.8%)	10.3 (93.2%)	
23 wards	7.8 (96.9%)	3.5 (43.8%)	4.8 (59.4%)	

During the state of emergency, each hospital restricted regular medical care systems to maintain their capability to treat COVID-19 cases. There was a major restriction of the number of outpatient departments in both the Tama area (−61.4%) and the 23 wards (−43.7%), and significant reductions of elective operations in the Tama area (−72.8%) and the 23 wards (−68.8%). After the lifting of the state of emergency, both numbers recovered quickly (*p* < 0.05). There was a rapid decrease in emergent stroke care. Compared to the Tama area, there were significant decreases in emergent operations in the 23 wards (−40.6%), and the trend of the restrictions continued to last. In particular, the acceptance of acute stroke cases by emergency transport in the 23 wards was also significantly decreased to <50% and remains <60%. In the Tama area, emergent operations and the acceptance of emergent acute stroke cases recovered to almost normal after the declaration was lifted.

## Comprehensive Stroke Center Score

**Figure 1:**
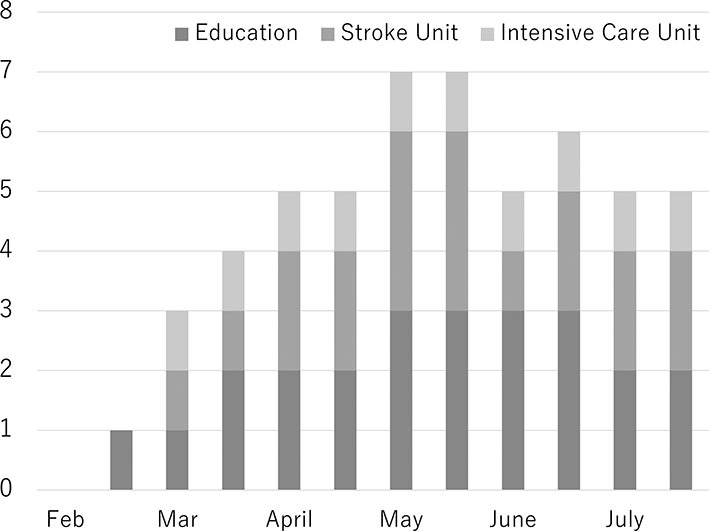
Accumulated number of items of comprehensive stroke center scores with deficits.

This score was assessed using 25 items divided into 5 components specifically recommended for CSCs. Personnel, diagnostic techniques, and specific expertise were not affected throughout this study. Infrastructure, including stroke units (maximum 3 hospitals) and intensive care units (1 hospital), was closed during this survey. Educational meetings were also not held (maximum 3 hospitals) (Figure 1).

## Discussion

The available preliminary data show lower volumes of acute stroke admissions, thrombolysis and MT cases in Tokyo over the first 6 months of the epidemic, including during the state of emergency. Each hospital restricted its medical care system, especially emergent operations and the acceptance of emergent acute stroke cases. The two main parts of the Tokyo metropolitan area, the Tama area and the 23 wards, were affected differently, probably due to the different numbers of COVID-19 cases.

Prior studies reported fewer thrombolysis and MT cases during the COVID-19 pandemic (6, 7), consistent with the present results in Japan. Fewer patients with TIAs sought hospital care, and the proportion of patients arriving within the therapeutic time window of thrombolysis was significantly lower (8). There are no reasons to assume that the incidence of stroke is decreasing. Some suggest the reason is that patients' fear of in-hospital infection causes avoidance behavior (1, 6, 8). In the present survey in Tokyo, it is noteworthy that MT rates, reflecting severe strokes, remained largely unaffected. This indicates that both patients and pre-hospital medical staff correctly recognize the need for urgent assessment and treatment despite the threat of COVID-19 infection.

The other findings of the present survey show the concrete restrictions of the medical care system in each hospital. Each hospital limited the acceptance of patients in the outpatient department and for elective operations first. With the increase in the number of COVID-19 patients, emergent operations and emergent stroke care systems were restricted to a major extent. The reason for the decrease of MT cases is not clear, whether it was a decrease in onset, the reluctance of patients, or a decrease in secondary transfer from regional stroke centers. The hospitals in the 23 wards were where the pandemic hit early and more severely (about 90% of the COVID cases in Tokyo), whereas the hospitals in the Tama area felt less impact of the COVID-19 pandemic, so that the difference in the extent of the effect may be mainly attributable to the lower number of COVID-19 cases in the Tama area. If an explosive increase of COVID-19 patients were to occur in the Tama area, an effect on the acute stroke care system similar to that seen in the 23 wards could easily occur.

Comprehensive stroke center score data were also collected. To the best of our knowledge, this is the first report of changes in CSC scores during the COVID-19 pandemic. It was found that some centers changed the structure of in-hospital stroke care during this pandemic. Resource management is critical during a pandemic. To date, there has been no explosive increase of COVID-19 patients in Japan. Each hospital restricted their infrastructure or educational activity according to their medical resources.

## Limitations

This was partly a retrospective study and not all thrombectomy-capable hospitals in the 23 wards cooperated. Data about stroke type, the delay to admission, time metrics from arrival to the hospital to the start of thrombolysis or to recanalization, and outcomes after reperfusion therapy were not collected. Whether the morbidity/mortality of stroke was increased in pandemic of COVID-19 as compared with normal situation has not become clear. Finally, one might not be able to extrapolate the results to other countries or regions with different stroke care protocols and different social and healthcare responses to the COVID-19 pandemic. The strength of the present survey is that it provides real-world information about stroke quality metrics in stroke centers in Japan.

## Conclusions

The COVID-19 pandemic has had a marked impact on stroke care in Tokyo, Japan. With various timely and appropriate changes to an institution's acute stroke care system, the medical care system must maintain its capacity to treat acute stroke patients to a similar extent as pre-pandemic. Further studies will need to confirm recent findings with a larger cohort, comparing stroke treatment time metrics and long-term outcomes between pre-pandemic and pandemic acute stroke patients.

## Data Availability Statement

The original contributions generated in the study are included in the article/supplementary materials, further inquiries can be directed to the corresponding author.

## Ethics Statement

The studies involving human participants were reviewed and approved by The Institutional Review Boar of each participating institute. Written informed consent for participation was not required for this study in accordance with the national legislation and the institutional requirements.

## Author Contributions

TO collected, analyzed, interpreted the data, and wrote the manuscript. YS and TH were major contributions in the study design and editing of the manuscript. All authors read and approved the final manuscript.

## Conflict of Interest

TH received honoraria from Daiichi-Sankyo, Nippon Boehringer Ingelheim, Bayer, Pfizer, Bristol Myers Squibb, and Medtronic. The remaining authors declare that the research was conducted in the absence of any commercial or financial relationships that could be construed as a potential conflict of interest.
